# Rickettsial Disease in the Peruvian Amazon Basin

**DOI:** 10.1371/journal.pntd.0004843

**Published:** 2016-07-14

**Authors:** Claudine Kocher, Amy C. Morrison, Mariana Leguia, Steev Loyola, Roger M. Castillo, Hugo A. Galvez, Helvio Astete, Carmen Flores-Mendoza, Julia S. Ampuero, Daniel G. Bausch, Eric S. Halsey, Manuel Cespedes, Karine Zevallos, Ju Jiang, Allen L. Richards

**Affiliations:** 1 Kantonsspital Baden, Baden, Switzerland; 2 U.S. Naval Medical Research Unit No. 6, Lima and Iquitos, Peru; 3 Instituto Veterinario de Investigaciones Tropicales y de Altura, Iquitos, Peru; 4 Instituto Nacional de Salud, Lima, Peru; 5 Laboratorio de Investigacion y Desarrollo, Universidad Peruana Cayetano Heredia, Lima, Peru; 6 U.S. Naval Medical Research Center, Silver Spring, Maryland, United States of America; University of Texas Medical Branch, UNITED STATES

## Abstract

Using a large, passive, clinic-based surveillance program in Iquitos, Peru, we characterized the prevalence of rickettsial infections among undifferentiated febrile cases and obtained evidence of pathogen transmission in potential domestic reservoir contacts and their ectoparasites. Blood specimens from humans and animals were assayed for spotted fever group rickettsiae (SFGR) and typhus group rickettsiae (TGR) by ELISA and/or PCR; ectoparasites were screened by PCR. Logistic regression was used to determine associations between patient history, demographic characteristics of participants and symptoms, clinical findings and outcome of rickettsial infection. Of the 2,054 enrolled participants, almost 2% showed evidence of seroconversion or a 4-fold rise in antibody titers specific for rickettsiae between acute and convalescent blood samples. Of 190 fleas (*Ctenocephalides felis*) and 60 ticks (*Rhipicephalus sanguineus*) tested, 185 (97.4%) and 3 (5%), respectively, were positive for *Rickettsia* spp. *Candidatus* Rickettsia asemboensis was identified in 100% and 33% of the fleas and ticks tested, respectively. Collectively, our serologic data indicates that human pathogenic SFGR are present in the Peruvian Amazon and pose a significant risk of infection to individuals exposed to wild, domestic and peri-domestic animals and their ectoparasites.

## Author Summary

Rickettsial infection remains relatively unexplored in South America compared to other regions of the world. For most regions of Peru (including the Amazon Basin), nothing more than broad serological characterization is available about circulating rickettsiae. Even less is known about the animal reservoirs and insect vectors involved in disease transmission. With this study we aimed to better characterize the circulating species of *Rickettsia* in humans in the Amazon Basin, as well as investigate their domestic animal reservoir and arthropod vectors. Out of 2054 fever patients enrolled we identified 38 individuals with serologic evidence for acute rickettsial infection. Their homes were visited in order to draw blood samples and collect ectoparasites from their domestic animals. Serology and molecular methods were used to test the animal blood samples as well as the ectoparasites. The information collected contributes to the understanding of the transmission dynamics of rickettsial diseases in Iquitos and leads to a better understanding of the exposure risk to rickettsial infection and it will guide approaches for prevention.

## Introduction

Rickettsiae and rickettsia-like organisms are a diverse group of obligate intracellular bacteria within the order Rickettsiales that include members of the genera *Rickettsia*, *Orientia*, *Ehrlichia*, *Anaplasma*, *Neorickettsia* and *Wolbachia*. Based on whole-genome analysis, species within the genus *Rickettsia* are divided into four geno-groups: 1) spotted fever group rickettsiae (SFGR), which comprises *R*. *rickettsii*, *R*. *conorii* and others; 2) typhus group rickettsiae (TGR) with *R*. *prowazekii and R*. *typhi*; 3) an ancestral group with the non-pathogenic members *R*. *bellii* and *R*. *canadensis;* and 4) a transitional group that harbours the disparate species *R*. *akari*, *R*. *australis* and *R*. *felis*. This latter group is often still included within the SFGR due to antigenic relatedness. Not all of the known *Rickettsia* species are pathogenic to humans.

Outbreaks caused by some *Rickettsia* species are occasionally associated with enzootic vectors, such as mosquitoes, fleas, mites and ticks [1–7]. Rickettsial diseases are neglected, potentially severe, but easily treatable and preventable. Due to overlapping symptoms, it is often not possible to distinguish them from dengue (and other arbovirus infections), leptospirosis, typhoid fever, malaria, or enterovirus infection. Overall, the distribution of rickettsial pathogens is poorly characterized in Peru with the exception of *R*. *parkeri*, a pathogen identified in *Amblyomma maculatum* ticks from northwestern Peru [8] and *Candidatus* Rickettsia andeanea [9], a novel rickettsial species detected during a febrile disease outbreak investigation in the town of Sapillica in northern Peru. Previous data from the Amazon Basin of Peru suggested that rickettsial agents represent potential causes of fever [10] and that seroprevalence in domestic animals was high [11]; however, there remains a paucity of information on risk factors, vectors and circulating rickettsial species.

Rickettsial infections remain underrecognized and underreported due to a lack of awareness and limited access to diagnostics, many of which are often suboptimal. Current research efforts are directed both towards defining the epidemiology of these diseases and development of improved diagnostics.

The objectives of our study were: (1) to determine the proportion of human febrile illnesses that were associated with rickettsial infections and (2) to identify vectors and reservoirs of rickettsial pathogens in the Peruvian Amazon.

## Materials and Methods

### Study site

This study was conducted in Iquitos, which is located in the Amazon forest (73.2’W longitude, 3.7°S latitude, 120 m above sea level) in the Department of Loreto, in the northeastern region of Peru. The city is populated by approximately 400,000 people and is accessible only by air or river [12]. Participants included in the study lived in neighborhoods in urban, peri-urban or rural Iquitos.

### Ethics statement

This project was nested in an ongoing surveillance study (protocol # NMRCD.2010.0010), which was approved by the Naval Medical Research Unit, No 6 (NAMRU-6) Institutional Review Board (Lima, Peru) in compliance with all U.S. federal regulations governing the protection of human subjects. In addition, the study protocol was reviewed and approved by health authorities in Peru (Instituto Nacional de Salud (INS), Direccion General de Epidemiologia (DGE) and Dirección Regional de Salud Loreto (DIRESA-LORETO)). Written informed consent was obtained from patients 18 years of age and older. For patients younger than 18 years, written informed consent was obtained from a parent or legal guardian. Additionally, written assent was obtained from patients between 8 and 17 years of age.

Animal handling and ectoparasite collection was approved and performed in accordance with the NAMRU-6 Animal Care and Use Committee (NAMRU-6 Protocol number 13–5). The experiments reported herein were conducted in compliance with with the Animal Welfare Act and in accordance with the principles set forth in the “Guide for the Care and Use of Laboratory Animals,” Institute of Laboratory Animals Resources, National Research Council, National Academy Press, 1996. Written informed consent was obtained from all animal owners before specimen collection.

### Human samples

The proportion of human febrile illness associated with rickettsial infection was determined by testing human samples obtained through an ongoing clinic-based passive febrile surveillance study. The febrile surveillance study offered enrollment if the individual presented to one of 12 health facilities (3 hospitals and 9 outpatient clinics) distributed across 4 districts of Iquitos; 2 were military health facilities and the rest were Ministry of Health hospitals and clinics. Febrile patients fulfilled the inclusion criteria of the surveillance study if their fever (axillary temperature ≥ 37.5°C) duration was ≤ 5 days and they were 5 years or older. A serum sample was collected at the time of enrollment (acute) and a convalescent serum sample was obtained 10–30 days later. These samples have already been used to investigate other pathogens (mainly dengue virus and other arboviruses); the results and testing methods are described in detail elsewhere [13,14].

### Animal and ectoparasite collections

The objective of identifying vectors and reservoirs of rickettsial pathogens was approached by a prospective sentinel-case driven, case-control design that was nested in the ongoing surveillance study for human febrile disease described above. We visited households of human participants whose laboratory results indicated recent rickettsial infection. We asked residents if they had contact with domestic animals such as dogs, cats, pet birds, and livestock (pigs, poultry and guinea pigs). If so, they were invited to participate in the study after a brief explanation of the study objectives and procedures. After obtaining permission from the owner of the domestic animals, we obtained blood samples and removed ectoparasites including (but not limited to) fleas, ticks and lice. The location, name and description of the animal and owner’s address were collected for each animal. In order to obtain a control-group of animals and ectoparasites, we randomly selected another household at a distance of greater than 500 m but under 2 km away from the sentinel household. Controls were processed as described for households with confirmed rickettsial disease. The ratio of rickettsial and non-rickettsial households was 1:1. Additionally, all military bases in and around Iquitos were visited and the same procedure was repeated there.

Depending on the size of the animal, we collected 1–3 ml of blood using EDTA tubes. Tubes were immediately stored on ice and transported to NAMRU-6‘s Iquitos field laboratory within 3 hours. Samples were centrifuged and plasma was separated and stored at -80°C until further testing. The residual blood cells were placed in a separate vial and immediately stored at -80°C.

Ectoparasites were collected from domestic animals using combs and tweezers and placed in vials that were dry, plastic, and covered. Fleas, ticks and lice from each animal were placed in separate vials (maximum 30 specimens per vial). The vials were stored on ice until transport to the Iquitos field laboratory, where fleas, ticks and lice were taxonomically identified and stored at -80°C until shipment on dry ice to Lima, Peru, for DNA extraction and molecular analysis. Fleas, ticks and lice were identified according to the entomological keys of Aragao and Fonseca (1961), Graham and Price (1997), and Johnson (1957) [15–17]. Ticks, fleas and lice were pooled by species and individual host animal. Prior to laboratory testing, ectoparasites were rinsed using distillated water, placed on a sterile petri dish and divided into 2 pieces using sterile surgical blades. A new blade was used for each ectoparasite. Ticks and lice were divided longitudinally, whereas fleas were cut horizontally, dividing upper from lower body part. Half of each ectoparasite was immediately frozen and stored, and the other half was used for DNA extraction.

### Laboratory testing

#### Serologic methods

We screened convalescent serum samples collected from participants enrolled between January 2013 through February 2014 by SFGR- and TGR-specific IgG ELISAs [18,19]. This in-house assay has been validated extensively on 3 continents [11,20,21]. For those samples that tested positive for anti-SFGR or–TGR IgG antibodies (minimum titer of 100), we then carried out a side-by-side end-point titrations of the acute and convalescent sera. We classified samples with a seroconversion or a 4-fold or greater increase in IgG antibody titer between acute and convalescent blood samples and minimum titer of 1:400 in the convalescent sample as indicative of recent active rickettsial infection [22].

The ELISA was performed using a *R*. *rickettsii* whole-cell antigen coated micro-well format. Ninety-six-well plates (Dynatech Laboratories, Chantilly, VA) were coated with a 1:4000 dilution of whole–cell antigen from *R*. *rickettsii* R strain in PBS, prepared as previously described [23]. For the assessment of *R*. *typhi* exposure, the same ELISA method was performed, except a whole-cell antigen from *R*. *typhi* Wilmington strain was used [19].

Animal plasma samples were tested using the same ELISA for SFGR as in human samples except a goat anti-dog, goat anti-cat, goat anti-duck, goat anti-chicken HRP conjugated antibodies (Kirkegaard & Perry Laboratories, Gaithersburg, MD) and a protein A/G peroxidase conjugated (from Thermo Fisher Scientific, Waltham, MA) were used instead of the goat anti-human HRP conjugated antibody. Positive and negative controls for each species tested were used (dog, cat, poultry).

### DNA extractions

DNA was extracted from human and animal whole blood samples using QIAmp DNA mini kits (QIAGEN, Valencia, CA) following the manufacturer’s instructions into a final elution volume of 100 μl and stored at -80°C. Ectoparasite halves were extracted individually by mechanical disruption using 100 μl of PrepMan Ultra Sample Preparation Reagent (Applied Biosystems, Waltham, MA) and a Kontes Pellet Pestle (Thermo Fisher Scientific, Waltham, MA). After grinding, individual samples were heated to 95°C for 10 minutes using a heat block. To clarify them, samples were centrifuged at room temperature, for 5 minutes at top speed (13,000 rpm) using a table top Eppendorf centrifuge (Hamburg, Germany). Cleared supernatants were transferred to clean tubes and stored at -20°C until further processing.

### PCR assays

Human and animal whole blood samples were individually screened using a qPCR assay targeting the *Rickettsia* genus-specific 17-kD gene (Rick17b) that has been previously described [24]. Positive (plasmid) and negative (no template control—water) controls were used in all the assays. To optimize the efficiency of workflows for ectoparasites, individually extracted samples were pooled by host in groups of 5 samples or less per pool. Pools were initially screened using Rick17b [24]. Samples in positive pools were further tested individually using the same approach. Individual positive samples were then tested using a nested PCR assay that also targeted the 17-kD gene but that can differentiate between SFGR and TGR [3]. Positives from this screen were then tested using two additional qPCR assays that targeted variable regions of the *ompB* gene: *R*. *felis* group (RfelG) and the *Ca*. Rickettsia asemboensis genotype (Rasemb) [3].

### Statistical analysis

Statistical analysis was performed using Stata 12 (StatCorp., College Station, TX, USA). Data were double entered and crosschecked. Means with corresponding standard deviations (SD) or medians and interquartile ranges are presented for normally and non-normally distributed variables, respectively, to account for the sampling design.

Comparisons across groups for categorical variables were done with Chi-square test or Fisher's exact test if an expected cell count was less than five. Continuous variables were analyzed with Student’s t-test. The association between seroconversion (recent active infection) and independent variables was determined using logistic regression. To evaluate strength of association, odds ratios and their 95% confidence intervals were calculated. Multivariate logistic regression was performed with seroconversion as the outcome, using substantive knowledge to guide variable selection. All variables that were associated with an outcome at a significance level of p<0.10 in the univariate analysis were included in the initial model. The significance level for removal from the model was set at p = 0.06 and that for addition to the model at p = 0.05. Strength of association was determined by estimating the odds ratio and the 95% confidence intervals (CI).

Logistic regression models were constructed with the dichotomous dependent variable SFGR or TGR seroconversion (recent active infection) to evaluate risk factors for infection. For those with evidence of active infection, symptoms and clinical findings as originally reported in the participant questionnaires were evaluated. We tested: age (three age categories), sex, occupation (4 categories: students, home-based, high-risk exposure, other), and potential animal contact (Table 1). The occupation groups were formed using the information on the participant questionnaire when available. “Home-based occupation” contains housewives, retired and unemployed individuals. The “High-risk exposure occupation” group contains active military duty (majority), local law enforcement and occupations outside of town such as hunting, fishing, farming and logging. The group of “others” contains various job activities, such as self-employed individuals, occupation in health establishments, office, construction or merchandising. The following independent variables were evaluated additionally as risk factors for acquiring an infection: trip outside of the city during 15 days prior to presentation at health care facility and contact with febrile individual 15 days prior to presentation. Symptoms, clinical findings and information on the course and outcome of disease (hospitalization, length of stay, duration of illness, disease evolution at follow-up visit) were also analyzed. The different bleeding manifestations (epistaxis, oral mucosal bleeding, hematochezia, hematuria and hematemesis) were collapsed to “any form of bleeding” due to the small sample size among seroconverters. The group of participants with evidence of co-infection with rickettsial agents and another pathogen were further analyzed separately (S1 Table).

**Table 1:** Univariate logistic regression analysis of age, occupation and other risk factors for active spotted fever group (SFGR) infection compared to infections due to other causes.

Variable	SFG seroconversion (n = 38)		Other febrile illnesses (n = 2016)		OR (95% CI)	*P* value
	No	%	No	%		
Age median (range)	25 (21–33)		23(17–36)		1.009	0.42
Age by categories						
Age < = 20	8		798		-	
Age 21–35	22		683		3.2 (1.4–7.2)	0.05
Age >35	8		535		1.5 (0.6–4)	0.427
Male sex	21	55.2	997	50.5	1.2 (0.63–2.30)	0.56
Occupation of total n = 2042						
Students	4 of 38	10.5	646 of 2004	32	0.25 (0.08–0.69)	0.008
High-risk exposure occupations	15 of 38	39.5	450 of 2004	21.1	2.25 (1.2–4.4)	0.016
Home-based occupations	13 of 38	34.2	485 of 2004	24.2	1.62 (0.83–3.2)	0.159
Other occupations	6 of 38	15.8	423 of 2004	21.1	0.7 (0.3–1.7)	0.428
Contact with febrile individual during the past 15 days	19 of 38	50	824 of 2014	41	1.44 (0.76–2.7)	0.26
Animal contact reported	29	76	1264	62.7	1.9 (0.9–4)	0.09
Trip outside of city during 15 days prior to presentation	6	15.8	209	10.4	1.62 (0.67–3.9)	0.284

## Results

### Febrile surveillance

Between January 2013 and February 2014, 2,562 participants were enrolled in the main study. Of them, 2,054 had paired (acute and convalescent) samples and therefore they became our study population. The median age of this population was 23 years and ranged from 5 to 82 years. The population was evenly distributed by gender, and nearly 20% were military. Of the 2,054 participants tested, 786 (37.4%) were infected with dengue virus when tested by PCR and/or isolation confirmed by indirect immunofluoresence assay. Other arboviruses were detected by PCR and/or isolation in 27 of cases (1.3%) (3 Mayaro virus, 1 Group C orthobunyavirus (only isolation), 1 Maguari virus, 22 Venezuelan equine encephalitis virus).

### Recent active rickettsial infection

When tested for TGR IgG no seroconversions or 4-fold rise of titer were detected. Thirty-eight (1.85%) of all febrile participants seroconverted or had a 4-fold or greater rise in titer of SFGR IgG between their acute and convalescent samples. These were classified as active rickettsial infections at the time of acute sample collection. Of these, 13 (34.2%) were identified in a military hospital, 12 (31.6%) were on active military duty, with 9 living permanently in camp. The active rickettsial infection cases were identified from 11 out of 12 study sites across and around the city.

Univariate logistic regression analysis indicated that people 21–35 years of age, being a student, and having a high risk occupation were risk factors for acute rickettsial infection (Table 1). Median age and sex did not differ between groups. Univariate logistic regression analysis of symptoms, clinical findings and course of febrile illness suggested that SFGR infection was associated with longer duration and persistence of illness at the follow-up visit (Tables 2 and 3). None of the analyzed symptoms and clinical findings were clearly associated with rickettsial infection. Only photophobia showed an association in univariate analysis, which did not remain statistically significant in multivariate analysis. In the multivariate logistic regression model, home-based occupations as well as high-risk occupations were associated with SFGR seroconversion (Table 4). The longer duration of illness remained significantly associated in multivariate analysis.

**Table 2:** Univariate logistic regression analysis of symptoms and clinical findings during infection with SFGR compared to other febrile illnesses.

Variable	SFGR seroconverters n = 38		Other febrile illnesses n = 2016		OR 95% CI	*P* value
	No	%	No	%		
Chills	38	100	1928	95.6	-	-
Headache	38	100	1999	98.1	-	-
Malaise	37	97.3	2004	99.4	0.22 (0.02–1.7)	0.153
Myalgia	35	92	1865	93	0.94 (0.3–3.1)	0.925
Generalized body pain	34	89.4	1876	93	0.63 (0.22–1.8)	0.39
Joint pain	33	87	1839	91.2	0.64 (0.2–1.6)	0.35
Retrooccular pain	33	87	1699	84	1.23 (0.5–3.1)	0.67
Anorexia	33	86.8	1854	91.7	0.58 (0.2–1.5)	0.26
Nausea	29	76.3	1540	76.4	0.99 (0.5–2.1)	0.99
Dizziness	28	73.7	1552	77	0.83 (0.4–1.7)	0.63
Abdominal pain	25	65.8	1358	67.3	0.93 (0.5–1.8)	0.84
Photophobia	22	58	777	38.5	2.19 (1.1–4.2)	0.018
Vomiting	18	47.3	876	43.4	1.17 (0.6–2.2)	0.63
Conjunctival injection	17	44.7	744	37	1.38 (0.7–2.6)	0.32
Rash	16	42.1	1036	51.4	0.69 (0.4–1.3)	0.26
Diarrhea	12	31.6	756	37.5	0.77 (0.4–1.5)	0.46
Itching	12	31.6	794	39.4	0.71 (0.4–1.4)	0.33
Otalgia	8	21	266	13	1.75 (0.8–3.9)	0.16
Cough	7	18.4	366	18.1	1.01 (0.4–2.3)	0.97
Rhinorrhea	5	13.2	319	15.8	0.8 (0.3–2)	0.66
Sore throat	5	13.1	392	19.4	0.63 (0.2–1.6)	0.34
Expectoration	4	10.5	259	12.9	0.8 (0.3–2.3)	0.67
Dyspnea	4	10.5	170	8.9	1.2 (0.4–0.01)	0.72
Any bleeding manifestations	1	2.6	234	11.6	0.2 (0.02–1.5)	0.12
Temperature, mean (SD) of total n = 2048	38.1 (0.89)		37.9 (0.86)		1.2 (0.9–1.8)	0.245
Tourniquet positive	15 of 38	39.5	651 of 1972	33	1.32 (0.7–2.6)	0.40

**Table 3:** Univariate analysis of the outcome of infection due to SFGR compared to other febrile illnesses.

Variable	SFGR seroconverters, n = 38		Other febrile illnesses, n = 2016		OR 95% CI	*P* value
	No	%	No	%		
Hospitalization	8	21	556	27.6	0.70 (0.3–1.5)	0.37
Mean duration of fever (SD, range in d)	4.3 (2.6, 1–14)		4.1 (1.9, 1–17)		1.04 (0.9–1.2)	0.62
Mean duration of illness (SD, range in d)	9.34 (6.1, 2–26)		7.4 (4, 1–30)		1.09 (1–1.2)	0.004
Mean duration of Hospitalization (SD, range in d)	4.1 (3.1, 1–8)		5.2 (2.9, 1–31)		0.82 (0.6–1.2)	0.258
Persistence of symptoms at follow-up visit	8	21	214	10	2.24 (1–4.9)	0.046

**Table 4:** Adjusted Odds-ratios based on multivariate logistic regression model of spotted fever group seroconverters (n = 38) compared to other febrile illnesses (n = 2016).

Variable	OR (95% CI)	*P* value
High-risk exposure occupation	3.7 (1.6–8.5)	0.001
Home-based occupation	2.4 (1–5.6)	0.042
Duration of illness	1.1 (1–1.1)	0.005

Of the 38 participants with serologic evidence of active rickettsial infection, 14 (36.8%) and 2 (5.2%) of the participants were co-infected with dengue virus and Venezuelan Equine Encephalitis virus, both diagnosed by PCR (S1 Table). The remaining 22 did not have serologic or molecular evidence of co-infection with an arbovirus. We also tested the 38 acute samples of the seroconverters by PCR targeting the 17-kD antigen, but none tested positive.

### Domestic animals

Domestic animals were sampled from 15 households of the 38 human participants with evidence of active rickettsial infection, that were eligible for a household visit. The remaining 23 participants and their households were not included for the following reasons: inaccessibility of the dwelling (10.5%), no animal contact (30.4%), participant had been transferred away from military camp (39.1%), refused participation (4.3%), could not be located (4.3%), and pet had died since febrile episode (4.3%). Overall, we visited 30 households (15 sentinel and 15 control households) and 5 military camps. During these visits, a total of 51 dogs, 9 cats and 14 other animals (1 pet bird, 1 duck, 4 goats and 8 chickens) were sampled.

We identified anti-SFGR IgG antibodies in only 3 animal samples: 1 cat (titer of 400) and 1 chicken (titer of 1600) from separate military camps; and 1 chicken from a sentinel household (titer of 400). All 51 animals tested were negative for the 17-kD antigen gene targeted by PCR.

### Ectoparasites

Of a total of 284 ectoparasites collected (Table 5): 190 were fleas from dogs and cats (all of which belonged to the species *Ctenocephalides felis*); 34 were lice from poultry and dogs (3 belonged to the species *Goniocotes gallinae*, 1 to *Menopon gallinae* and the rest to *Menacanthus stramineus*); and 60 were ticks from dogs (all of which belonged to the species *Rhipicephalus sanguineus*). All samples were tested for the presence of rickettsia using a variety of PCR assays. Initially, all 284 samples were tested using Rick17b qPCR assay. Of these, 188 (185 fleas and 3 ticks) were positive and 96 were negative. This result was confirmed using an additional 17KDa nested PCR assay that distinguished between the SFGR and TGR. With this assay, we determined that all 188 positives belonged to the SFGR. Further testing (RfelG *R*. *felis*-genogroup specific qPCR assay and *ompB* fragment (2484-bp) sequencing) allowed us to determine that the 188 positives contained *Rickettsia felis*-like organisms, but none were positive for *Rickettsia felis*. Out of these 188 positive samples 79 (76 fleas and 3 ticks) were further tested with the recently described *Ca*. Rickettsia asemboensis species-specific Rasemb qPCR assay and all 76 fleas as well as 1 of 3 ticks were positively identified as *Ca*. R. asemboensis. The two ticks negative for the Rasemb qPCR assay contained *R*. *felis*-like organisms not *R*. *felis* or *Ca*. R. asemboensis.

**Table 5:** Overview of ectoparasites collected.

	*Ctenocephalides felis*	*Goniocotes gallinae and Menopon gallinae*	*Menacanthus stramineus*	*Rhipicephalus sanguineus*
Total	190	4	30	60
Collected from dogs	161	-	5	60
Collected from cats	29	-	-	-
Collected from poultry	-	4	25	-
Positive by qPCR and/or nested PCR (17-kD), n (%)	185 (97.4%)	0 (0%)	0 (0%)	3 (5%)
Positive for *R*. *felis*-like organisms	185 of 185 tested			3 of 3 tested
Positive for *R*. *asemboensis*	76 of 76 tested			1 of 3 tested

## Discussion

We demonstrated that almost 2% of individuals presenting with a febrile episode in Iquitos had evidence of recent active SFGR infection when serologic testing was performed during a 14-month period. TGR infection did not seem to play an important role in causing human infection in this area though 1.0% of febrile patients had evidence of a previous infection with TGR. This proportion of febrile cases due to SFGR infection is very similar to previous studies [25]. However, in that previous study the sample size was much smaller. In particular, our study is the first that systematically analyzed febrile samples for rickettsial infection in the area of Iquitos, Peru.

We showed that among study participants with evidence of rickettsial infection, the presentation of disease was non-specific and symptoms or clinical findings did not help guide diagnosis. This is in agreement with previous reports where rickettsial infection presented as acute febrile illness with poor clinical predictors [26,27]. Further detailed comparison of febrile patients with SFGR infection to those without SFGR did not reveal any helpful differences [28,29] and diagnosis was mostly made retrospectively. This underlines the inability to rely on clinical presentation and rapid reliable diagnostic methods to identify cases. In our study we defined severe disease by respiratory distress, circulatory collapse, multiorgan failure, loss of consciousness, fluid accumulation or shock; having any of these manifestations was rare among febrile patients (3 patients) and was not identified among patients with rickettsial infection. But 21% of patients with rickettsial infection were hospitalized with a mean duration of 3.1 days (range 1 to 8 days). During this study we did not record if the patients received antibiotic treatment during the hospitalization or if a rickettsial infection was suspected in this context by the treating physician. But it is not uncommon in this setting for the patients to receive doxycycline as other diseases considered in the differential diagnosis such as leptospirosis are also endemic in this study area. During the study follow-up convalescent visit, significantly more patients with rickettsial disease reported persistent symptoms than those with another febrile disease. This impact of morbidity was also demonstrated by significantly longer duration of illness reported for rickettsial infection compared to the other febrile patients.

According to the analysis of patient data, we demonstrated that acute rickettsial infection was strongly associated with home-based occupations (including housewives, retired and unemployed individuals) but also with rural and out of town (high-risk) exposure occupations, while other professions were not associated with the disease. The majority of those working in high-risk exposure professions were military personnel, and most of them lived permanently on base and were men. All military individuals affected in our study worked extensively outdoors. The protective effect of being a student in the univariate analysis is probably confounded by age and thus appropriately fell out of our final multivariate model.

Sixteen of our participants with evidence of active rickettsial infection presented with a co-infection with an arbovirus. This is an important finding, as rickettsial infection can be treated with antibiotics (i.e., doxycycline); however, it would not be uncommon for a physician in a dengue endemic area to stop looking for additional infections in someone with presumed or confirmed dengue. The high percentage of co-infections among those with rickettsial infection could raise concerns about the specificity of our in house ELISA assay; however, the observation that out of 786 dengue virus positive samples (by PCR and/or isolation), only 14 showed seroconversion to SFG argues against this being a non-specific reaction. Also, co-infection causing rickettsial disease plus another illness (such as dengue, malaria or other bacterial diseases) has been well-demonstrated [29,30]. Unfortunately, our study could not determine which pathogen—arbovirus (mainly dengue virus), rickettsial agent, or both—was responsible for the observed clinical symptoms. In a separate analysis of the co-infection subgroup, we observed statistically significant features distinguishing co-infections from either mono-infected arboviral or monoinfected rickettsial infections (S1 Table), but the very low number of co-infections precludes drawing definitive conclusions. The substantial proportion of co-infections may represent the similarity of epidemiological risk factors for the different infections.

We were unable to confirm the diagnosis of rickettsial infection in our study with specific molecular diagnostic assays. This is not surprising considering the low sensitivity when performed in blood samples [22]. PCR, culture or immunohistochemical identification using biopsies of skin lesions (rash or eschar) would be desirable and is reported to have much higher sensitivity [22,31,32] but is not available in many limited-resource settings and was not part of our study procedure. This underlines the fact that empiric anti-rickettsial treatment should often be based on the clinicoepidemiologic diagnosis due to the retrospective nature of the currently available serologic tools.

Surprisingly, reported contact with animals was not associated with active rickettsial infection. In accordance, our evaluation of domestic animals did not reveal that they played a major role as hosts for rickettsial pathogens of the SFGR. This finding contrasts with the results of a previous study in this area where almost 60% of all dogs tested were found to be positive for SFGR antibodies [11]. Besides differences in sample size and location of animals tested, the different serological methods used could explain some of the differences observed. Unfortunately, we could not detect rickettsial pathogens by PCR in any of the animal samples. This could possibly support the conclusion that domestic animals do not seem to be an important reservoir for rickettsiae; however, sensitivity with animal blood samples is known to be low and varies with the molecular methods used [33], as is the case with human specimens [22]. Another limitation of this study was the fact that we did not sample rodents. In our data, home-based occupations were a clear risk factor for contracting the infection, which indicates exposure to a reservoir in and around the house. At the same time, reporting a high-risk profession, which mainly represents living on a military camp was also associated with rickettsial infection. Both occupation groups could have exposure to rodents.

By analyzing the ectoparasites collected from domestic animals, we demonstrated that almost all of the fleas were positive for *Ca*. R. asemboensis, which belongs to the SFGR. However, the known rickettsial flea-borne pathogens, *R*. *typhi* and *R*. *felis* were not detected. *Ca*. R. asemboensis was originally isolated from fleas from Kenya [34] and has recently been reported in Ecuador [35]. The high prevalence of *Ca*. R. asemboensis in fleas known to bite humans and to transmit agents that cause human illness gives rise to suspect this agent could be transmitted to humans. However, in a similar situation in Kisumu, Kenya, where high prevalence of *Ca*. R. asemboensis was found, only *R*. *felis* DNA was found in febrile patients blood [20]. At this time, this agent’s involvement as a cause of human disease cannot be ruled out. The involved flea species (*C*. *felis*) is known to bite a variety of animals, including rodents. Collecting rodent samples in and around human housing would be an important next step in order to investigate the presence of both the SFGR and TGR infections.

In conclusion, almost 2% of all undifferentiated febrile illnesses in Iquitos, the major Peruvian city in the Amazon basin, had an active rickettsial infection based on serology. Similar to other past reports, we could not identify features that would distinguish rickettsial diseases from other endemic diseases, and thus permit implementation of appropriate treatment. We demonstrated that home-based occupations, as well as high-risk occupations outside of town, were risk factors for rickettsial infection. This implies that exposure to domestic, as well as non-domestic animals could be important. In those individuals with undifferentiated fever, clinicians should be aware of the possibility of co-infection with rickettsial pathogens, even in confirmed arboviral cases as our study demonstrated. This has direct consequences for management and treatment and can potentially impact morbidity and time missed from work. Although hospitalization rate and severity of disease did not differ between rickettsial disease and other febrile illnesses, the association with prolonged duration of illness can have an important impact on morbidity and health care cost. Evaluation of specimens from domestic animals and their ectoparasites revealed a high percentage of fleas infected with *Ca*. R. asemboensis. Human pathogenicity of *Ca* R. asemboensis and its main reservoir remains to be determined.

## Supporting Information

S1 Table Univariate analysis comparing participants with co-infections to those without (DOCX)
