# Supplementary material for: Rickettsial Disease in the Peruvian Amazon Basin
Source: PLoS Negl Trop Dis. 2016 Jul 14;10(7):e0004843. doi: 10.1371/journal.pntd.0004843 (PMC4944934; doi:10.1371/journal.pntd.0004843)
Supplement: S1 Table — (DOCX) [file pntd.0004843.s001.docx]

| S1 Table. Univariate analysis comparing participants with co-infections to those without | | | | | | | | | | | | | | |
| --- | --- | --- | --- | --- | --- | --- | --- | --- | --- | --- | --- | --- | --- | --- |
| Variable | Patients with co-infections  n=16 | | | Patients with only acute rickettsial infection n=22 | | | OR (95% CI) | P value | Patients with only acute arboviral infection n=779 | | | OR (95% CI) | | P value |
|  | | N | % | | N | % |  |  | | N | % |  |  | |
| Age median (range) | | 22 (18-31) |  | | 27 (22-36) |  | 0.95 (0.9-1) | 0.154 | | 22 (16-32) |  | 1.0 (1-1.04) | 0.99 | |
| Male sex | | 13 | 81 | | 8 | 36 | 7.58 (1.6-35) | 0.009 | | 403 | 52 | 4.0 (1.1-14.3) | 0.030 | |
| Occupation: | | |  | |  |  |  |  | |  |  |  |  | |
| Students | | 3 | 18.8 | | 1 | 4.6 | 1.0 |  | | 288 of 775 | 37 | 1.0 |  | |
| Home-based occupation | | 2 | 12.5 | | 11 | 50 | 0.1 (0.004-0.9) | 0.043 | | 170 of 775 | 22 | 1.1 (0.2-6.8) | 0.895 | |
| High-risk exposure occupation | | 7 | 43.8 | | 8 | 36.4 | 0.3 (0.02-3.5) | 0.330 | | 153 of 775 | 19.7 | 4.4 (1.1-17.2) | 0.034 | |
| Others | | 4 | 25 | | 2 | 9.1 | 0.7 (0.04-11.3) | 0.779 | | 164 of 775 | 21.2 | 2.3 (0.5-10.6) | 0.269 | |
| Other exposure | | |  | |  |  |  |  | |  |  |  |  | |
| Travel out of town during past 15d | | 3 | 18.8 | | 3 | 13.6 | 1.46 (0.3-8.4) | 0.67 | | 67 | 8.6 | 2.45 (0.7-8.8) | 0.17 | |
| Contact with febrile person (past 15d) | | 7 | 43.8 | | 12 | 54.6 | 0.65 (0.2-2.4) | 0.512 | | 296 of 778 | 38 | 1.27 (0.5-3.4) | 0.64 | |
| Animal contact reported | | 12 | 75 | | 17 | 77.3 | 0.88 (0.2-4) | 0.87 | | 504 | 64.7 | 1.64 (0.5-5.1) | 0.4 | |
| Presentation of symptoms and clinical findings | | | | |  |  |  |  | |  |  |  |  | |
| Variable | Patients with co-infections  n=16 | | | Patients with only acute rickettsial infection n=22 | | | OR (95% CI) | P value | Patients with only acute arboviral infection n=779 | | | OR (95% CI) | | P value |
|  | | N | % | | N | % |  |  | | N | % |  |  | |
| Chills | | 16 | 100 | | 22 | 100 | - | - | | 742 | 95.3 | - | - | |
| Malaise | | 16 | 100 | | 21 | 95.5 | - | - | | 774 | 99.4 | - | - | |
| Headache | | 16 | 100 | | 22 | 100 | - | - | | 773 | 99.2 | - | - | |
| Anorexia | | 16 | 100 | | 17 | 77.3 | - | - | | 730 | 93.7 | - | - | |
| Dysgeusia | | 14 | 88 | | 20 | 91 | 0.7 (0.09-5.6) | 0.736 | | 694 | 89 | 0.86 (0.2-3.8) | 0.84 | |
| Myalgia | | 14 | 87.5 | | 21 | 95.5 | 0.33 (0.03-4) | 0.39 | | 725 | 93 | 0.52 (0.1-2.4) | 0.397 | |
| Nausea | | 13 | 81.3 | | 16 | 72.7 | 1.625 (0.3-7.8) | 0.544 | | 628 | 80.6 | 1.0 (0.3-3.7) | 0.95 | |
|  | |  |  | |  |  |  |  | |  |  |  |  | |
| Dizziness | | 13 | 81 | | 15 | 68 | 2 (0.4-9.5) | 0.37 | | 633 | 81.3 | 10.3-3.6) | 0.99 | |
| Retro-occular pain | | 13 | 81 | | 20 | 90 | 0.433 (0.1-3) | 0.39 | | 672 | 86.3 | 0.69 (0.2-2.5) | 0.57 | |
| Joint pain | | 12 | 75 | | 21 | 95 | 0.143 (0.01-1.4) | 0.098 | | 715 | 91.8 | 0.27 (0.1-0.9) | 0.026 | |
| Conjunctival injection | | 10 | 62.5 | | 7 | 31.8 | 3.57 (0.9-14) | 0.065 | | 293 | 37.6 | 2.76 (1-7.7) | 0.051 | |
| Abdominal pain | | 9 | 56.3 | | 16 | 72.7 | 0.48 (0.1-1.9) | 0.29 | | 538 | 69 | 0.58 (0.2-1.6) | 0.28 | |
| Rash | | 9 | 56 | | 7 | 31.8 | 2.76 (0.7-10.5) | 0.14 | | 532 | 68.3 | 0.6 (0.2-1.6) | 0.31 | |
| Photo-phobia | | 9 | 56 | | 13 | 59 | 0.89 (0.2-3.3) | 0.86 | | 341 | 43.8 | 1.65 (0.6-4.5) | 0.32 | |
| Otalgia | | 7 | 43.8 | | 1 | 4.6 | 16.3 (1.7-153) | 0.014 | | 97 | 12.5 | 5.5 (2-15) | 0.001 | |
| Itching | | 7 | 43.8 | | 5 | 22.7 | 2.64 (0.6-10.8) | 0.174 | | 410 | 52.6 | 0.7 (0.3-1.9) | 0.48 | |
| Generalized body pain | | 14 | 41.1 | | 20 | 90.9 | 0.7(0.1-5.6) | 0.74 | | 719 | 92.3 | 0.58 (0.1-2.6) | 0.48 | |
| Vomiting | | 6 | 37.5 | | 12 | 54.6 | 0.50 (0.1-1.9) | 0.301 | | 406 | 52.1 | 0.55 (0.2-1.5) | 0.25 | |
| Diarrhea | | 4 | 25 | | 8 | 36.3 | 0.58 (0.1-2.4) | 0.46 | | 306 | 39.3 | 0.52 (0.2-1.6) | 0.26 | |
| Rhinorrhea | | 4 | 25 | | 1 | 4.5 | 7.0 (0.7-70) | 0.098 | | 67 | 8.6 | 3.51.1-11.3) | 0.032 | |
| Sore throat | | 4 | 25 | | 1 | 4.6 | 7.0 (0.7-70) | 0.098 | | 106 | 13.6 | 2.11(0.7-6.7) | 0.2 | |
| Cough | | 4 | 25 | | 3 | 13 | 2.1 (0.4-11) | 0.378 | | 97 | 12.5 | 2.3 (0.7-7.4) | 0.147 | |
| Expec-toration | | 3 | 18.8 | | 1 | 4.6 | 4.8 (0.5-52) | 0.191 | | 62 | 8 | 2.7(0.7-9.6) | 0.13 | |
| Dyspnea | | 1 | 6.3 | | 3 | 13.6 | 0.42 (0.04-4.5) | 0.47 | | 86 | 11 | 0.54 (0.1-4.1) | 0.55 | |
| Hemat-emesis | | 1 | 6.3 | | 0 |  | - | - | | 30 | 3.9 | 1.66 (0.2-13) | 0.63 | |
| Tempera-ture, mean (SD) | | 38.1 (0.8) |  | | 38.1 (1) |  | 1.08 (0.5-2.2) | 0.84 | | 38 (0.84); of n=777 |  | 1.18 (0.7-2.1) | 0.59 | |
| Tourniquet pos. | | 10 of 16 | 62.5 | | 5 of 22 | 22.7 | 5.67 (1.4-23.5) | 0.017 | | 315 of 762 | 41.3 | 2.37 (0.9-6.6) | 0.099 | |
| Burden of symptoms | | | | |  |  |  |  | |  |  |  |  | |
| Variable | Patients with co-infections  n=16 | | | Patients with only acute rickettsial infection n=22 | | | OR (95% CI) | P value | Patients with only acute arboviral infection n=779 | | | OR (95% CI) | | P value |
|  | | N | % | | N | % |  |  | | N | % |  |  | |
| Number of clinical symptoms, mean (SD; range) | | 16 (4.5) |  | | 14.3 (3.5) |  | 1.12 (0.9-1.3) | 0.206 | | 15.6 (3.5) |  | 1.0 (0.9-1.2) | 0.677 | |
| Outcome of infection | | | | |  |  |  |  | |  |  |  |  | |
| Mean duration of fever (SD) | | 3.7 (1.6) |  | | 4.7 (3) |  | 0.83 (0.6-1.1) | 0.253 | | 4.3 (1.7) |  | 0.78 (0.6-1.1) | 0.141 | |
| Mean duration of illness | | 11 (7.5) |  | | 8.1 (4.7) |  | 1.08 (1-1.2) | 0.165 | | 7.8 (4.2) |  | 1.1 (1-1.2) | 0.006 | |
| Hospitalization | | 3 | 18.8 | | 5 | 22.7 | 0.78 (0.2-3.9) | 0.767 | | 304 | 39 | 0.36 (0.1-1.3) | 0.114 | |
| Mean duration of Hospitalization (SD) | |  | 3.3 (4.0) | |  | 4.6 (2.9) | 0.86 (0.5-1.4) | 0.56 | | 5.2 (2.6) |  | 0.62 (0.3-1.2) | 0.151 | |
| Persistence of symptoms at follow-up visit | | 5 | 62.5 | | 11 | 36.7 | 2.88 (0.6-14.4) | 0.199 | | 86 | 11 | 3.66 (1.2-10.8) | 0.019 | |
